# Spatial distribution of elements during osteoarthritis disease progression using synchrotron X-ray fluorescence microscopy

**DOI:** 10.1038/s41598-023-36911-w

**Published:** 2023-06-23

**Authors:** Xiwei Fan, Kah Meng Lee, Michael W. M. Jones, Daryl Howard, Antonia Rujia Sun, Ross Crawford, Indira Prasadam

**Affiliations:** 1 Centre for Biomedical Technologies, School of Mechanical, Medical and Process Engineering, Queensland University of Technology, 60 Musk Ave/Cnr. Blamey St, Kelvin Grove, QLD 4059 Australia; 2 Central Analytical Research Facility, Queensland University of Technology, Brisbane, 4059 Australia; 3 School of Chemistry and Physics, Queensland University of Technology, Brisbane, 4000 Australia; 4 Australian Synchrotron, Melbourne, 3168 Australia; 5 The Prince Charles Hospital, Brisbane, 4032 Australia

**Keywords:** Imaging, Osteoarthritis

## Abstract

The osteochondral interface is a thin layer that connects hyaline cartilage to subchondral bone. Subcellular elemental distribution can be visualised using synchrotron X-ray fluorescence microscopy (SR-XFM) (1 μm). This study aims to determine the relationship between elemental distribution and osteoarthritis (OA) progression based on disease severity. Using modified Mankin scores, we collected tibia plates from 9 knee OA patients who underwent knee replacement surgery and graded them as intact cartilage (non-OA) or degraded cartilage (OA). We used a tape-assisted system with a silicon nitride sandwich structure to collect fresh-frozen osteochondral sections, and changes in the osteochondral unit were defined using quantified SR-XFM elemental mapping at the Australian synchrotron's XFM beamline. Non-OA osteochondral samples were found to have significantly different zinc (Zn) and calcium (Ca) compositions than OA samples. The tidemark separating noncalcified and calcified cartilage was rich in zinc. Zn levels in OA samples were lower than in non-OA samples (P = 0.0072). In OA samples, the tidemark had less Ca than the calcified cartilage zone and subchondral bone plate (P < 0.0001). The Zn–strontium (Sr) colocalisation index was higher in OA samples than in non-OA samples. The lead, potassium, phosphate, sulphur, and chloride distributions were not significantly different (P > 0.05). In conclusion, SR-XFM analysis revealed spatial elemental distribution at the subcellular level during OA development.

## Introduction

Osteoarthritis (OA) is a chronic degenerative joint condition affecting 528 million individuals worldwide. It is characterised by symptoms such as pain, stiffness, and swelling, and is known to cause significant disability, reduced quality of life, and comorbidities. Despite its widespread occurrence and profound impact on society, the exact causes of OA remain elusive. Therefore, expanding our knowledge of the disease mechanisms and exploring innovative therapeutic approaches to address this significant health challenge is urgently needed.

**Figure 1:**
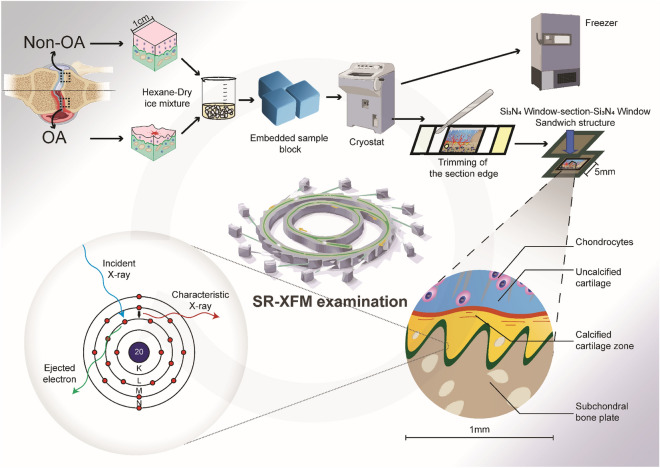
Illustrated workflow to analyse the spatial elemental distribution. Subsequent to collecting samples from total knee replacements, the samples were trimmed into 1 cm × 1 cm × 1 cm blocks utilising the EXAKT bandsaw and were plunge-frozen in a hexane-dry ice mixture. The samples were then embedded with SCEM and completely frozen in a hexane-dry ice mixture. Following the Kawamoto technique, the sample blocks were sectioned, and then medial OA and lateral non-OA sections were collected via a cryofilm tape-assisted system. The cryofilm tapes were subsequently trimmed using a surgical scalpel blade to fit the dimensions of Si_3_N_4_ windows. Lastly, the sections were embedded in a sandwich structure for SR-XFM analysis, and the figure was generated with BioRender.com.

The osteochondral interface is an essential component in the study of OA progression, as it is increasingly acknowledged as an active remodelling centre in the disease process. This interface is a thin interlayer between the hyaline articular cartilage, which provides a smooth surface for joint movement, and the subchondral bone plate (SBP), a compact bone supporting the cartilage. When examined under a microscope, the osteochondral interface is visible as a layer located between the tidemark (TM) and the cement line^1–4^ (Fig. 1). Within the osteochondral interface, the calcified cartilage zone (CCZ) is a mineralised tissue that plays a crucial role in maintaining joint health. It serves four primary functions in a healthy joint: (1) transmitting mechanical loading, (2) integrating bone and cartilage layers^5,6^, (3) helping the crosstalk between cartilage and bone^7,8^, (4) as well as transporting molecules^9–16^. In biological systems, the synchronisation of elements is indispensable for optimal tissue functioning. In particular, the distribution of mineral elements within the osteochondral interface greatly influences the biomechanical properties of the bone-cartilage unit, allowing for optimal joint function^2,17,18^. Furthermore, the intricate balance and interplay of different elements contribute significantly to the structural integrity, mechanical properties, cellular activities, and tissue repair processes. For example, Zinc (Zn) and Calcium (Ca) are crucial in maintaining joint stability, preserving mechanical properties, acting as co-factors in signalling pathways, and participating in vital biological activities during pathophysiological processes^19^. The element Strontium (Sr) exhibits significant similarities to the chemical attributes of Ca, making it another vital constituent found in the inorganic mineral accumulation at the osteochondral interface^20^. Potassium (K) contributes to molecular homeostasis through its involvement in membrane potential, electrolyte balance, pH regulation, enzymatic reactions, and cell growth^21^. Magnesium (Mg) and potassium (K) intake has been shown to have disease-modifying effects in OA^22,23^. Phosphate (P) is essential for bone and CCZ, forming hydroxyapatite and supporting energy metabolism as an ATP component while aiding in nucleic acid and coenzyme synthesis and acid–base balance^24^. Intra-articular basic calcium phosphate (BCP) crystals, present in most OA joints, are associated with severe degeneration^25^. Sulphur (S) is pivotal in tissue stability, contributing to amino acids, protein synthesis, redox balance, antioxidant protection, and synthesis of coenzymes and vitamins^26^. Chloride (Cl) has diverse electrolyte and acid–base balance functions, osmotic pressure regulation, nerve function, and digestion^27^. Dysfunction of Cl channels in articular cartilage can disrupt the microenvironment, leading to imbalances in the matrix and bone metabolism, partial aseptic inflammation, and progression of OA^28^. Additionally, certain heavy metals, including lead (Pb) and Cesium (Cs), may impede joint homeostasis through regional deposition^29,30^. In line with these studies, in our previous studies, we employed EDS and LA-ICP-MS as analytical tools to qualitatively identify the elemental composition in OA disease progression encompassing Ca, P, Sr, oxygen, carbon, K, Mg, Na, and Cl. However, due to the limited sensitivity and lack of spatial analysis to osteochondral interface and quantitative data of EDS and LA-ICP-MS, a more comprehensive investigation is warranted to acquire invaluable insights into the underlying mechanisms of the disease, which could ultimately lead to the development of innovative diagnostic tools and therapeutic strategies.

A range of methods have been devised for visualising the elemental composition and distribution in biological samples, such as Energy-dispersive X-ray spectroscopy (EDS), time-of-flight secondary ion mass spectrometry (TOF-SIMS), and laser ablation inductively coupled plasma mass spectrometry (LA-ICP-MS). These techniques are chosen based on spatial resolution, scanning speed, tissue-specific affinity, and penetration depth. Among different available methods, synchrotron X-ray fluorescence microscopy (SR-XFM) offers subcellular spatial resolution (with an effective pixel size of 1.0 μm depending on various parameters). It is a non-destructive method capable of imaging large tissue areas (mm^2^) with adequate penetration depth and minimal tissue preparation^31,32^. Despite its advantages, SR-XFM has not been used to study the endogenous distribution changes of osteochondral interface elements during OA due to challenges in preparing chemical-free fresh-frozen sections. Prior studies^33,34^ have employed resin-embedded samples for SR-XFM preparation, but the formalin or ethanol fixation during this process may alter the endogenous elemental distribution in cells^35^, soft tissues^36^, and hard tissues^37^; consequently, this could introduce bias and present an inaccurate picture of elemental distribution during disease progression^38^. To accurately examine endogenous changes, we developed a tape-assisted system that accommodated fresh-frozen tissue for SR-XFM^39^ and implemented a three-stage approach to improve data accuracy and value, which involved enhancements in sample preparation, image acquisition, and signal processing. This research aims to provide a comprehensive understanding of the spatial distribution and potential variations of elements within the osteochondral interface during OA progression. Specifically, we have choose to analyse the spatial distribution of Zn, Ca, Sr, Pb, K, P, S, Cl and Cs based on our previous studies because of their role in cartilage and joint homeostasis, The utilization of synchrotron X-ray fluorescence microscopy (SR-XFM) will enable a more detailed analysis, offering valuable insights into the underlying mechanisms of OA and paving the way for the development of innovative diagnostic tools and therapeutic strategies.

## Results

In order to examine the distribution patterns of elements in osteochondral tissues classified by the severity of the disease, we assessed the presence of Zn, Ca, Sr, Pb, K, P, S, Cl, and Cs in nine patient-matched osteochondral tissue sections based on the previous LA-ICP-MS and EDS results from our research group^40^, adhering to the process illustrated in (Fig. 1). The detailed modified Mankin score comparison is shown in Table 1.

**Table 1:** Modified Mankin score comparison.

	Non-OA	OA
Cartilage structure	1.96 ± 0.87	5.26 ± 0.54
Cellularity	0.95 ± 0.63	4.95 ± 0.98
Proteoglycan depletion	0.68 ± 0.73	1.91 ± 0.75
Tidemark integrity	–	0.99 ± 0.63
Total Mankin score	3.59 ± 0.66	13.13 ± 0.66

### Spatial distribution of Zn during OA progression

**Figure 2:**
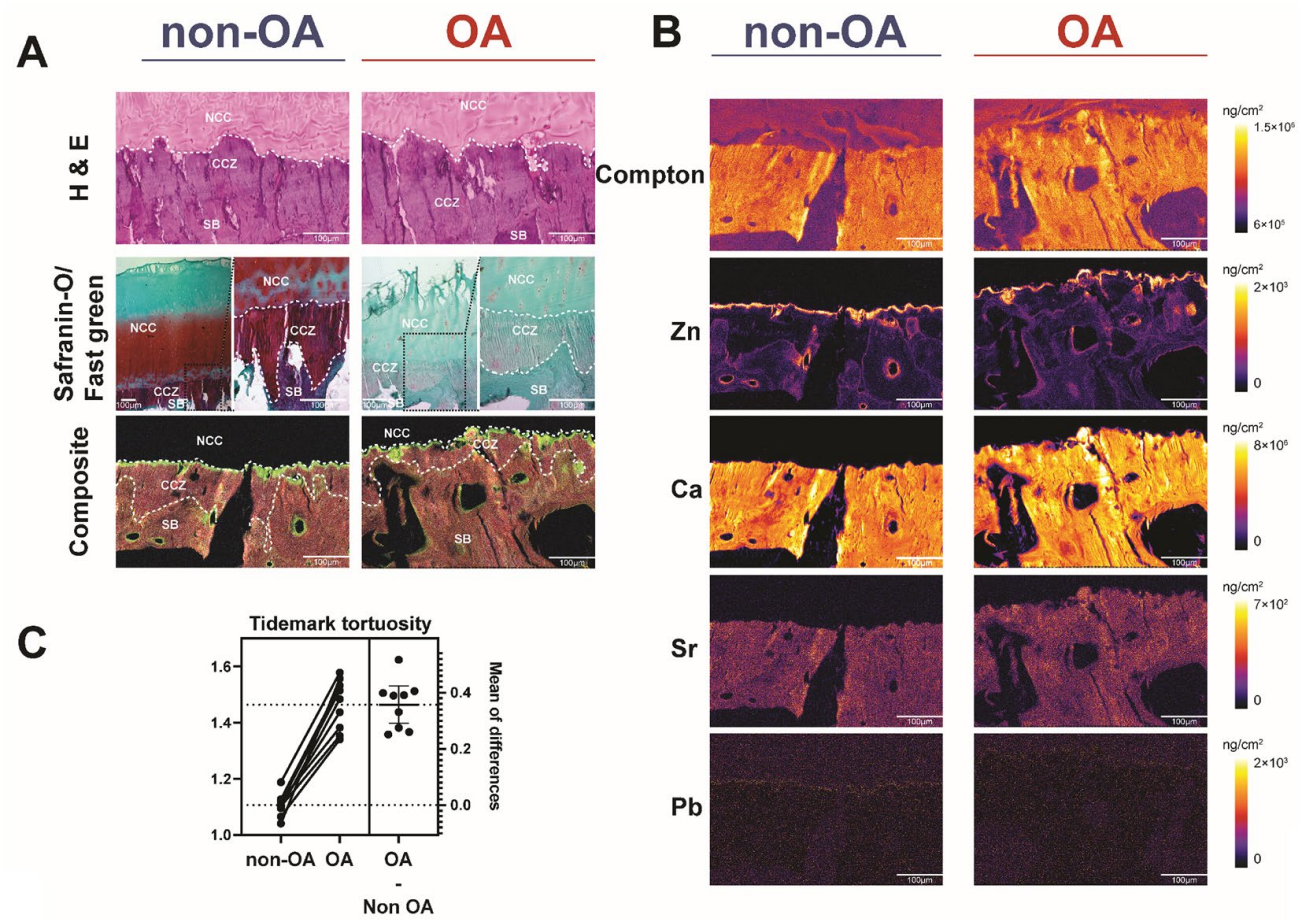
Elemental mapping of non-OA and OA osteochondral interface. (**A**) Representative elemental mapping of structural changes between non-OA and OA osteochondral interface. Figures are representative of n = 9 patient-matched samples. (**B**) Quantitative elemental osteochondral interface heatmaps in OA tissues graded according to disease severity. Representative Zn, Ca, Sr, and Pb elemental mapping of quantitative changes between non-OA and OA osteochondral interface. Figures are representative of n = 9 patient-matched samples. Scale bar: 100 μm. (**C**) Quantitation of the tidemark tortuosity index (length of the upper and bottom contour of tidemark from the starting to the endpoint). *NCC* Noncalcified cartilage, *CCZ* Calcified cartilage zone, *SBP* Subchondral bone plate, *TM* Tidemark. (n = 9, paired two-tailed t-test, data was presented as ng/cm^2^).

**Figure 3:**
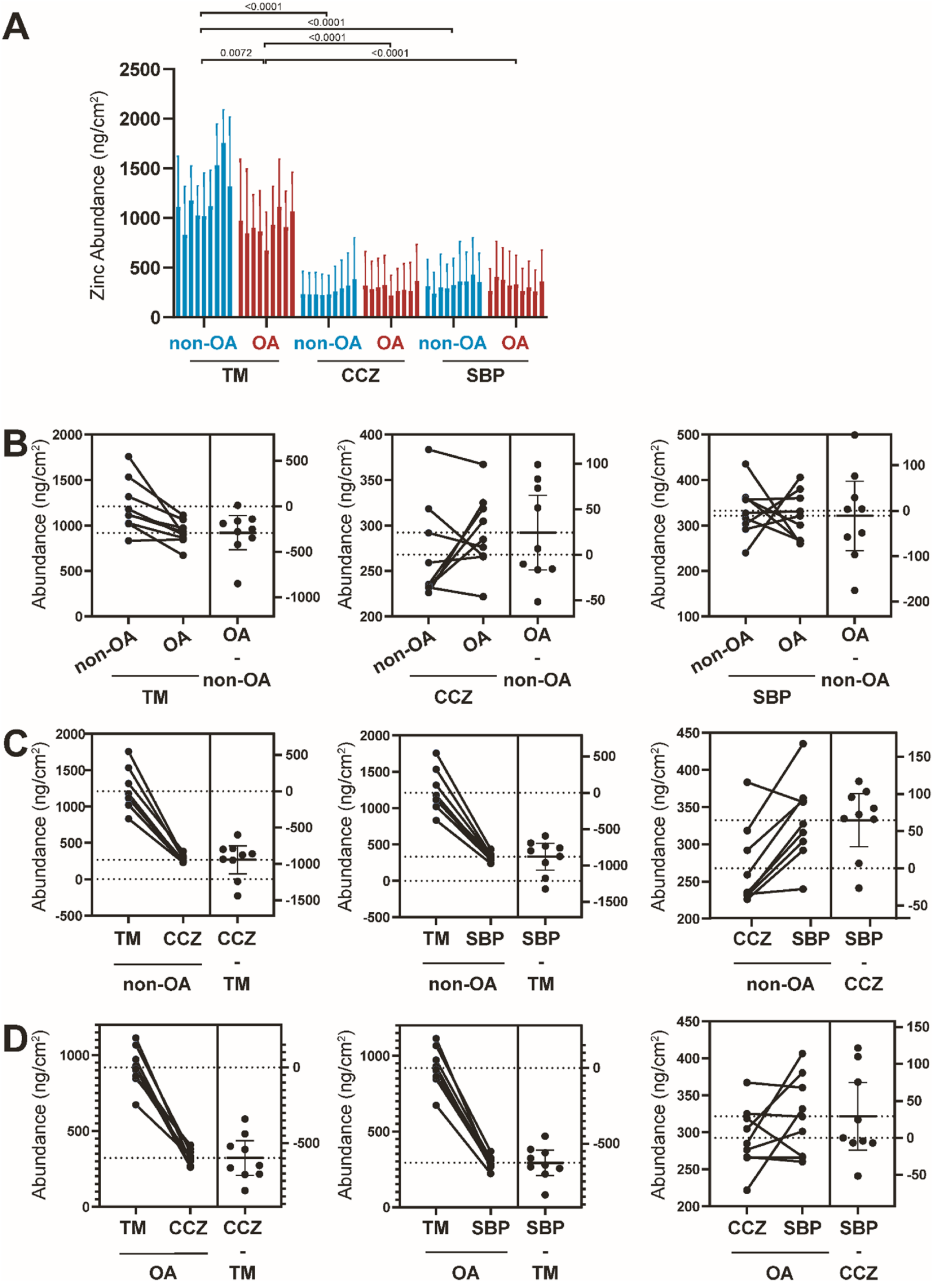
Quantitative Zn elemental changes of osteochondral interface graded according to disease severity. (**A**) The bar graph shows the Zn abundance using the technical replicates for each pixel derived from the region of interest (ROI) in individual specimens from non-OA and OA osteochondral sections. Values are expressed as Mean ± SD (data were gathered from each pixel including each region of interest, paired two-tailed t-test, data was presented as ng/cm^2^). (**B**) Paired T-test to compare group-wise differences in OA samples compared with non-OA samples. (**C**) Paired T-test to compare group-wise differences according to stratigraphy in non-OA samples. (**D**) Paired T-test to compare group-wise differences according to stratigraphy in OA samples. Figures are representative of n = 9 patient-matched samples. *TM* Tidemark, *CCZ* Calcified cartilage zone, *SBP* Subchondral bone plate. Values are expressed as Mean ± SD (n = 9, paired two-tailed t-test, data was presented as ng/cm^2^).

First, Zn accumulation was stratigraphically localised in the non-OA samples in the osteochondral junction. The TM region of the non-OA sample showed a strong Zn accumulation signal, followed by the SBP zone and CCZ zone (Fig. 2A,B). Quantitatively, Zn abundance for the non-OA for TM, CCZ, and SBP is 1210.70 ± 284.61.97 ng/cm^2^, 267.88 ± 53.85 ng/cm^2^, and 332.39 ± 54.82 ng/cm^2^, respectively. Additionally, the cement line, which is the region between SBP and CCZ, has a distinct high Zn-rich contour in non-OA samples. We noticed an intriguing high Zn accumulation in the chondrocyte lacunae in the non-OA CCZ region. In terms of osteochondral junction stratigraphy for OA, Zn localisation was more prevalent in TM, with Zn levels in OA samples being comparable between CCZ and SBP. Quantitatively, Zn abundance for OA TM, CCZ, and SBP is 919.45 ± 128.93 ng/cm^2^, 292.30 ± 42.11 ng/cm^2^, and 321.51 ± 53.03 ng/cm^2^, respectively. When comparing non-OA and OA samples, Zn is more abundant and wavier in the non-OA samples than in the OA sample in the TM area (P = 0.0072). Zinc follows the TM contour, and we found the zinc tortuosity index is increased in the OA sample (P < 0.0001, Fig. 2C). However, no difference was observed between CCZ and SBP when comparing non-OA and OA samples (P > 0.05, Fig. 3A–D).

### Spatial distribution of Ca during OA progression

**Figure 4:**
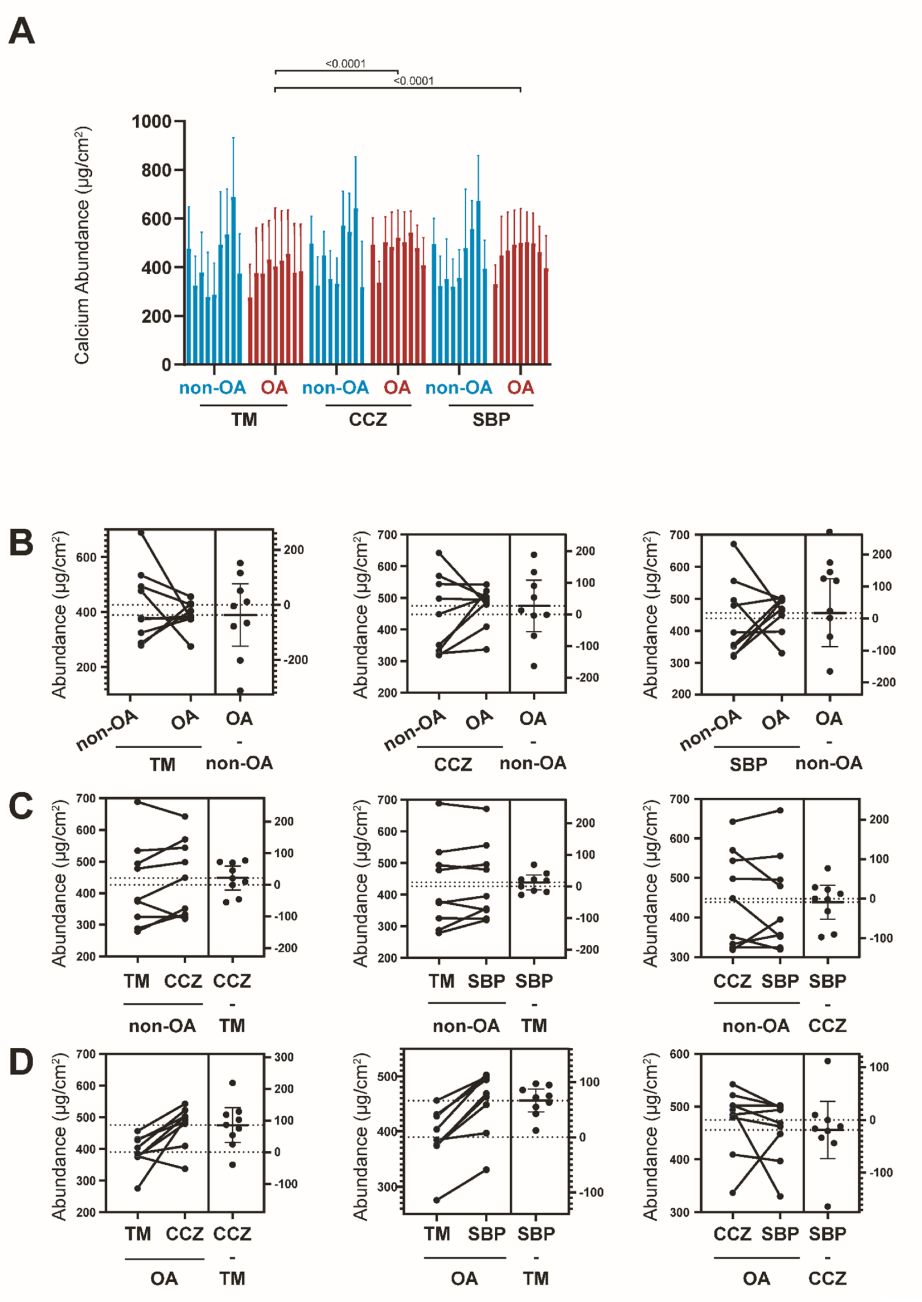
Quantitative Ca elemental changes of osteochondral interface graded according to disease severity. (**A**) The bar graph shows the Ca abundance using the technical replicates for each pixel derived from the region of interest (ROI) in individual specimens from non-OA and OA osteochondral sections. Values are expressed as Mean ± SD (data were gathered from each pixel including each region of interest, paired two-tailed t-test, data was presented as μg/cm^2^). (**B**) Paired T-test to compare group-wise differences in OA samples compared with non-OA samples. (**C**) Paired T-test to compare group-wise differences according to stratigraphy in non-OA samples. (**D**) Paired T-test to compare group-wise differences according to stratigraphy in OA samples. Figures are representative of n = 9 patient-matched samples. *TM* Tidemark, *CCZ* Calcified cartilage zone, *SBP* Subchondral bone plate. Values are expressed as Mean ± SD (n = 9, paired two-tailed t-test, data was presented as μg/cm^2^).

The Ca abundance varies depending on the osteochondral stratigraphy, similar to Zn. Both non-OA and OA samples' CCZ and SBP contain the same abundance of Ca (Fig. 2A,B). We observed an even Ca distribution throughout the non-OA TM with no distinction between TM, CCZ, and SBP observed. Quantitatively, the Ca abundance for non-OA TM, CCZ and SBP are 426.24 ± 134.08 μg/cm^2^, 447.57 ± 121.97 μg/cm^2^, and 438.46 ± 120.59 μg/cm^2^, respectively. The Ca content of OA TM is lower than that of OA CCZ and OA SBP (P < 0.0001 for both). In OA samples, we found no statistical difference in CCZ and SBP (P > 0.05). Quantitatively, the Ca abundance for OA TM, CCZ, and SBP is 389.44 ± 51.74 μg/cm^2^, 474.71 ± 63.35 μg/cm^2^, and 455.821 ± 58.05 μg/cm^2^, respectively. No statistical change was observed in TM, CCZ and SBP when comparing Ca abundance in patient-matched samples between the non-OA and OA counterparts (*P* > 0.05, Fig. 4A–D).

### Sr distribution changes during OA progression

**Figure 5:**
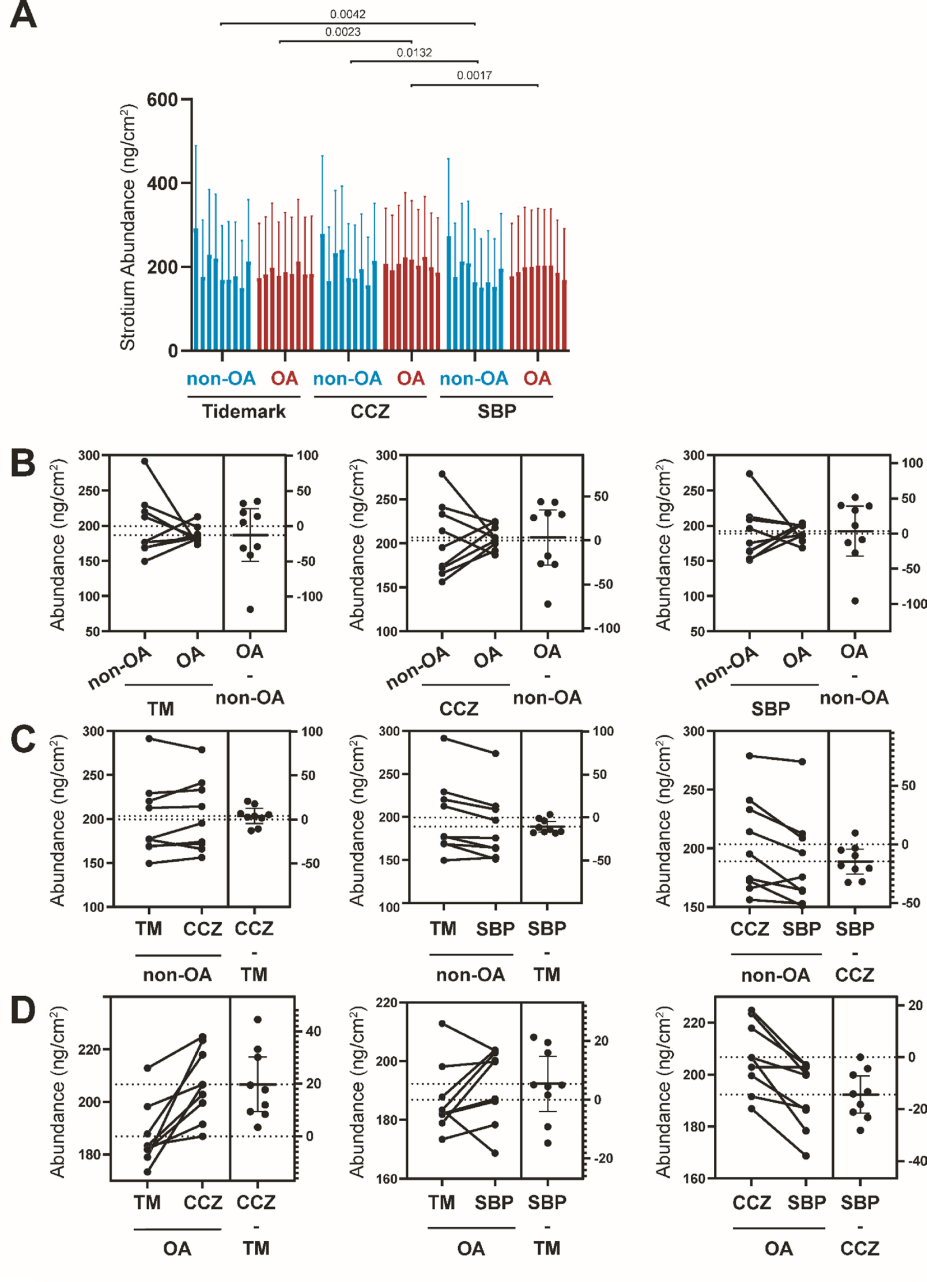
Quantitative Sr elemental changes of osteochondral interface graded according to disease severity. (**A**) The bar graph shows the Sr abundance using the technical replicates for each pixel derived from the region of interest (ROI) in individual specimens from non-OA and OA osteochondral sections. Values are expressed as Mean ± SD (data were gathered from each pixel including each region of interest, paired two-tailed t-test, data was presented as ng/cm^2^). (**B**) Paired T-test to compare group-wise differences in OA samples compared with non-OA samples. (**C**) Paired T-test to compare group-wise differences according to stratigraphy in non-OA samples. (**D**) Paired T-test to compare group-wise differences according to stratigraphy in OA samples. Figures are representative of n = 9 patient-matched samples. *TM* Tidemark, *CCZ* Calcified cartilage zone, *SBP* Subchondral bone plate. Values are expressed as Mean ± SD (n = 9, paired two-tailed t-test, data was presented as ng/cm^2^).

Sr follows a similar distribution pattern as Ca (Fig. 2A,B). The Sr abundance for non-OA TM, CCZ, and SBP is 199.41 ± 43.77 ng/cm^2^, 203.41 ± 41.30 ng/cm^2^, and 188.75 ± 45.21 ng/cm^2^, respectively. In non-OA samples, Sr distribution is lower in the SBP region compared with the TM (*P* = 0.0042) and the CCZ (*P* = 0.0132). The Sr abundance for OA TM, CCZ, and SBP is 186.87 ± 11.83 ng/cm^2^, 206.67 ± 13.33 ng/cm^2^, and 192.31 ± 12.77 ng/cm^2^, respectively. In OA samples, CCZ shows higher abundance compared with the TM (*P* = 0.0023) and SBP (*P* = 0.0017). When comparing non-OA and OA samples, the Sr abundance shows no statistical changes between the non-OA and OA counterparts (P > 0.05, Fig. 5A–D).

### Pb distribution changes during OA progression

**Figure 6:**
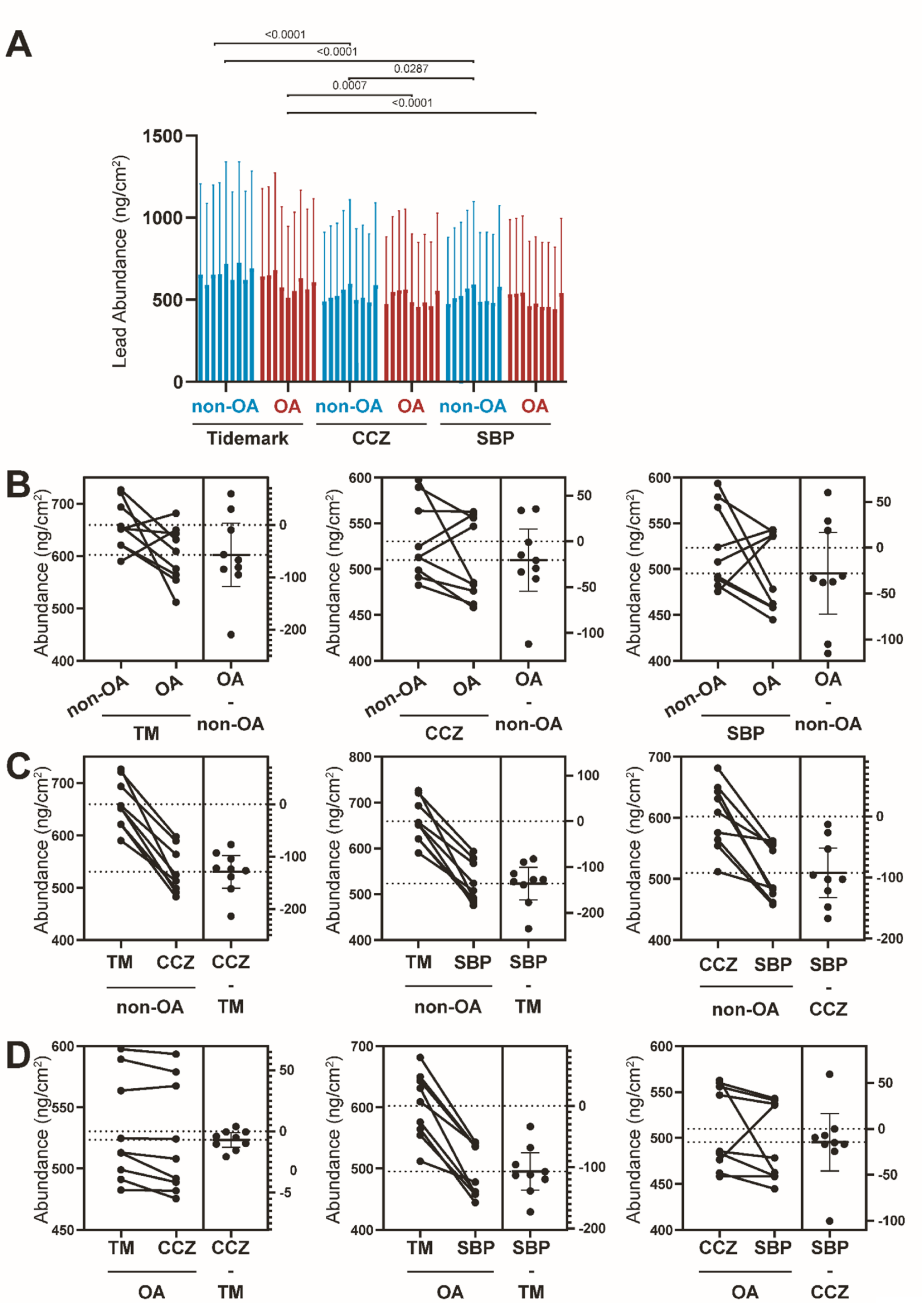
Quantitative Pb elemental changes of osteochondral interface graded according to disease severity. (**A**) The bar graph shows the Pb abundance using the technical replicates for each pixel derived from the region of interest (ROI) in individual specimens from non-OA and OA osteochondral sections. Values are expressed as Mean ± SD (data were gathered from each pixel including each region of interest, paired two-tailed t-test, data was presented as ng/cm^2^). (**B**) Paired T-test to compare group-wise differences in OA samples compared with non-OA samples. (**C**) Paired T-test to compare group-wise differences according to stratigraphy in non-OA samples. (**D**) Paired T-test to compare group-wise differences according to stratigraphy in OA samples. Figures are representative of n = 9 patient-matched samples. *TM* Tidemark, *CCZ* Calcified cartilage zone, *SBP* Subchondral bone plate. Values are expressed as Mean ± SD (n = 9, paired two-tailed t-test, data was presented as ng/cm^2^).

In terms of Pb distribution, we found Pb accumulation in the TM region (Fig. 2A,B), consistent with previous research^34^. The Pb abundance for non-OA TM, CCZ, and SBP is 659.35 ± 46.60 ng/cm^2^, 530.41 ± 42.67 ng/cm^2^, and 523.41 ± 45.22 ng/cm^2^, respectively. The abundance of Pb was higher in TM than the SBP in both non-OA (*P* < 0.0001). Non-OA CCZ showed higher Pb abundance than non-OA SBP (P = 0.0287). The Pb abundance for OA TM, CCZ, and SBP is 602.39 ± 54.50 ng/cm^2^, 509.99 ± 45.04 ng/cm^2^, and 495.40 ± 42.45 ng/cm^2^, respectively. OA TM shows higher Pb abundance compared with SBP (P < 0.0001). When comparing non-OA and OA samples, the Pb abundance shows no statistical changes (P > 0.05, Fig. 6A–D).

We found no statistical difference in the spatial distribution of other elements, such as K, P, S, and Cl, between non-OA and OA samples (P > 0.05). The phenomenon is due to XFM's inherent higher sensitivity for metal elements, particularly from Titanium to Uranium, but lower sensitivity for lighter elements (Fig. S1).

### Colocalisation of elements during OA progression

Next, we used colocalisation analysis to reveal the co-distribution changes of elements during OA progression. The results showed that Ca–Sr, Ca–Zn, and Zn–Sr colocalisation index is higher in TM than CCZ and SBP in both non-OA and OA samples (*P*_Ca–Sr_ = 0.032; *P*_Ca–Zn_ = 0.001 < ; *P*_Zn–Sr_ = 0.00151, Figs. S2–S4). OA Zn-Sr in the TM has a higher slope than non-OA (P = 0.015 < 0.05, Fig. S4). In both non-OA and OA samples, the Pb–Sr colocalisation index was higher in TM than CCZ and SBP (*P* = 0.017 < 0.05, Fig. S5), while no correlation was found between Ca–Pb samples (*P* > 0.05, Fig. S6). No correlation was found between Zn–Pb in both non-OA and OA samples (*P* > 0.05, Fig. S7).

## Discussion

To our knowledge, this is the first paper that reports changes in the stratigraphy and disease-specific differences of elements in OA graded according to disease severity, in which the lateral side is healthy, and the medial side is damaged based on modified Mankin score system at a subcellular resolution (1 μm)^33,34^.

In the present work, we found that unique spatial patterns of element distribution exist at the osteochondral interface. According to statistical analyses, non-OA osteochondral samples differ significantly from OA osteochondral samples in their elemental compositions, especially for Zn, Ca, and Pb. The TM separating the calcified cartilage from the noncalcified cartilage showed a significant Zn level. Unexpectedly, the Zn content of the OA TM was lower than that of the NON-OA TM (P = 0.0072). Additionally, we discovered less Ca in the TM in the OA samples than in the subchondral bone plate and calcified cartilage zone (P < 0.0001 for both). The Zn-Sr colocalisation index was higher in the OA TM region than in the non-OA samples.

TM has traditionally been regarded as a remnant of the growth plate during secondary ossification. Prior studies have identified alterations in the TM, such as increased tortuosity index, TM duplication, and endochondral ossification occurring during the progression of OA^40,41^. These changes have been linked to the redifferentiation of chondrocytes in the CCZ^42^. Discontinuity of the TM at the osteochondral interface has also been observed during OA progression^40,41,43,44^. However, these studies mainly provided descriptive analyses without quantitative data. Our findings suggest that in non-OA samples, TM discontinuity is closely associated with an uneven distribution of Zn in the TM region. Additionally, a higher Zn-Sr colocalisation index indicates a distinct regional variation in Zn distribution.

We also noticed a substantial change in Zn abundance and distribution in the TM area between non-OA and OA samples. Additionally, we found that the tortuosity index, shaped by Zn, is significantly increased (Fig. 2A). An increase in TM tortuosity is a characteristic identified through Safranin-O/fast green staining and is often associated with the reactivation of endochondral calcification and bone remodelling^40,44^. During biological processes, Zn interacts with various enzymes, such as alkaline phosphatase (ALP) and matrix metalloproteinases (MMPs), and serves as an essential trace element in numerous enzymes' reactive cores, contributing to healthy skeletal growth^45^. Although the precise role of Zn in bone metabolism remains unclear, recent studies suggest that it promotes bone formation by enhancing osteoblastic cell proliferation^46^. Conversely, research on Zn-deficient rats found no differences in bone mineral density, turnover, architecture, or biomechanics compared to control subjects^47^. In light of these findings, we employed laser microdissection microscopy for proteomics identification in the CCZ and identified several Zn-related proteins, including superoxide dismutase (SOD1), S100 calcium-binding protein A7 (S100A7), ALP, and others^40^. The Zn change could also be related to crystal formation change, as researchers found a higher mineral crystal thickness in the lateral compartment of OA^48^. However, no evidence exists that the Zn contributed to the crystal growth at this stage; further study is needed to elucidate the specific mechanism behind Zn accumulation in the TM.

This research also discovered a higher concentration of Ca in the CCZ of OA joints compared to the TM. The CCZ is an area of active remodelling during OA progression. Earlier studies have shown that the thickness of both the CCZ and the SBP undergoes dynamic changes during OA development^40,44^. In the early stages, CCZ thickness increases while SBP thickness decreases, but this reverses in later stages. These thickness fluctuations lead to active mineral changes within the CCZ and SBP. The exact role of abnormal mineralisation in the interaction between CCZ and SBP is still unclear. Additionally, it is well-established that collagen fibres and mineralisation determine the stiffness of the CCZ and SBP^40^. Our previous research found a reduction in the elastic modulus of both the CCZ and SBP, but the present study did not find any significant differences in Ca levels^40^. This discrepancy may be due to the combined effects of collagen bundles and the mineralisation process on the elastic modulus of the samples. A prior study reported increased stiffness in the collagen fibres of osteoarthritic cartilage, which could explain the observed changes in the elastic modulus^49^. As OA progresses, the reorganisation and entanglement of collagen fibres inevitably lead to a decrease in the elastic modulus.

Blood Pb levels are commonly recognised as a risk factor for knee OA^29^. However, the impact of regionally deposited Pb on OA progression is poorly understood. We discovered Pb accumulation in the deeper layers of the TM (TM). It is important to note that the process of Ca^2+^ being replaced by Pb^2+^ in Ca-hydroxyapatite is well-established at high Pb concentrations and is expected to occur similarly at low concentrations^50^. However, our study revealed that Pb does not have the same distribution as Ca, as it exhibits a higher affinity for the TM area, which is consistent with prior reports^33,34^. Earlier studies have suggested that Pb accumulation may contribute to disease progression^33,34^. However, we found no correlation between Pb and other elements in the osteochondral interface, nor between non-OA and OA samples. Our patients were recruited from Brisbane in Australia, a city with a consistently good air quality index (PM 2.5 ranging from 10 to 30). This low exposure to Pb might explain why it does not reach a level that can influence or accelerate OA progression. Future studies analysing local Pb accumulation in the tidemark and its relation to OA progression may help clarify the association.

There are some limitations to the current study. All the samples were collected from OA patients undergoing knee replacement surgery. Therefore, some changes may be overlooked or underestimated compared to normal samples without the signs of OA. The stratification of the non-OA and OA samples could omit the dynamic changes in the moderate OA stage, which will be investigated in future studies. Another limitation is that different elements hold different thresholds when performing quantitative XFM^51^. Therefore, some light elements have lower sensitivity, so we cannot measure the difference. However, this does not mean there is no difference between different groups.

This study presents novel findings regarding the changes in stratigraphy and disease-specific differences of osteoarthritis (OA) elements at a subcellular resolution. We observed unique spatial patterns of element distribution at the osteochondral interface and significant differences in elemental compositions between non-OA and OA osteochondral samples, highlighting the significance of elemental distribution in OA pathogenesis.

## Methods

### Human ethics and sample preparation

All methods were performed in accordance with guidelines and regulations, which were approved by the ethics committee of the Queensland University of Technology (Human ethics number: #1400001024). Nine participants provided written informed consent to donate the tissues for the knee arthroplasty surgery. After the surgery, the medial and lateral bearing surfaces of the tibia plates of the human donors were collected from St Vincent's Private Hospital. Based on the Modified Mankin scoring system^52^, the samples from each patient were matched and graded as non-OA (Grade 0–1) and OA (Grade 4) samples by three blinded observers, in which non-OA is the relatively intact knee joint with cartilage and SBP. In contrast, OA contains degraded cartilage and SBP sclerosis (Detailed demographic data is shown in Table S1). Patients with inflammatory bone diseases were excluded from the study. Patients with a previous medical history, bisphosphonate, and other medication therapy that could contribute to bone and cartilage metabolism and the elemental change were also excluded. EXAKT 310 Diamond Band Saw (EXAKT Apparatebau GmbH & Co. KG; Norderstedt, Germany) was used to cut off the intact and lesion part of the cartilage, and 1 cm × 1 cm × 1 cm cubes were trimmed. We plunge-froze samples in a hexane-dry ice mixture which were then embedded in the super cryo embedding medium (SCEM) (SECTION-LAB, Japan) and froze completely in the hexane-dry ice mixture using the Kawamoto technique^53^.

### Sectioning

Following our previously published protocol^39^, we sectioned all samples at 10 μm thickness using a CryoStar NX70 cryostat (ThermoFisher Scientific, USA) with a tungsten carbide knife, D profile (Dorn & Hart Microedge, USA). Then, since better sections were produced at a lower temperature^54^, we set the specimen at – 30 °C and the knife at – 28 °C. After that, we attached the tissue to Kawamoto's cryofilm tapes (3C(16UF), SECTION-LAB, Japan) to support the tissue and cut blocks into 5 mm × 5 mm pieces. We flipped over the tape (with sections on top) and mounted them on a Si_3_N_4_ window (600 nm thick; Australian National Fabrication Facility, QLD, Australia) with the matching tissue face exposed for analysis. After that, we freeze-dried the windows and stored them at room temperature in a sealed container to avoid protein degradation. We randomly positioned the samples in the frame by an observer-blind method to avoid selection bias.

### X-ray fluorescence microscopy image quantification

Prior to the beamtime, we mapped to identify the osteochondral interface using optical and fluorescence mosaic of the windows obtained using both Olympus VS120 Slide scanner and Zeiss LSM 710 Confocal Laser Scanning/Multi-photon Microscope with OlyVIA 2.9 software. Fluorescent mapping was undertaken at ~ 15 keV, with 5 mm × 5 mm areas mapped per sample at low resolution. We selected representative regions of interest with 1 mm × 1 mm size from these mappings. Next, we scanned these regions at high resolution and high sensitivity at parameters to achieve the best possible elemental maps. XFM photons were gathered at the Australian Synchrotron's XFM beamline as an event mode data stream^55^ using the Maia detector system^56^ and processed using the dynamic analysis method^57^ as implemented in GeoPIXE^58^. The data were quantified using well-characterised metallic foils and exported as 32-bit tiffs with units in areal density (ng**/**cm^2^). The tortuosity index was described as the ratio of the meandering curve to the straight-line length between the endpoints^59^.

### Colocalisation analysis

Following the previously published protocol^60^, we measured Zn, Ca, Pb, and Sr elemental mappings using colocalisation analysis as Pearson's r, Costes' regression threshold, and Mander's overlap coefficients, performed in Fiji using the 'Coloc 2' plugin.

### Statistical analysis

GraphPad Prism 8 (San Diego, USA) software was used to compute statistical analysis. They grouped experimental replicate data from each group and calculated the mean values at the sample level for further statistical comparison. A Shapiro–Wilk test was performed to assess the normality of the data, and all tested data groups passed this test successfully. After that, a paired t-test was conducted, and statistical significance was defined as p-values less than 0.05 for the above procedures. The researchers reported all data as mean values along with their standard deviation (SD).

## Supplementary Information

Supplementary Figures.

Supplementary Table S1.

## Data Availability

The manuscript contains all the necessary data. Any remaining information can be obtained from the corresponding author upon reasonable request.
