# Supplementary material for: Spatial distribution of elements during osteoarthritis disease progression using synchrotron X-ray fluorescence microscopy
Source: Sci Rep. 2023 Jun 23;13:10200. doi: 10.1038/s41598-023-36911-w (PMC10290122; doi:10.1038/s41598-023-36911-w)
Supplement: Supplementary file 1 — Supplementary Figures. [file 41598_2023_36911_MOESM1_ESM.docx]

**SUPPLEMENTARY FIGURES**

**
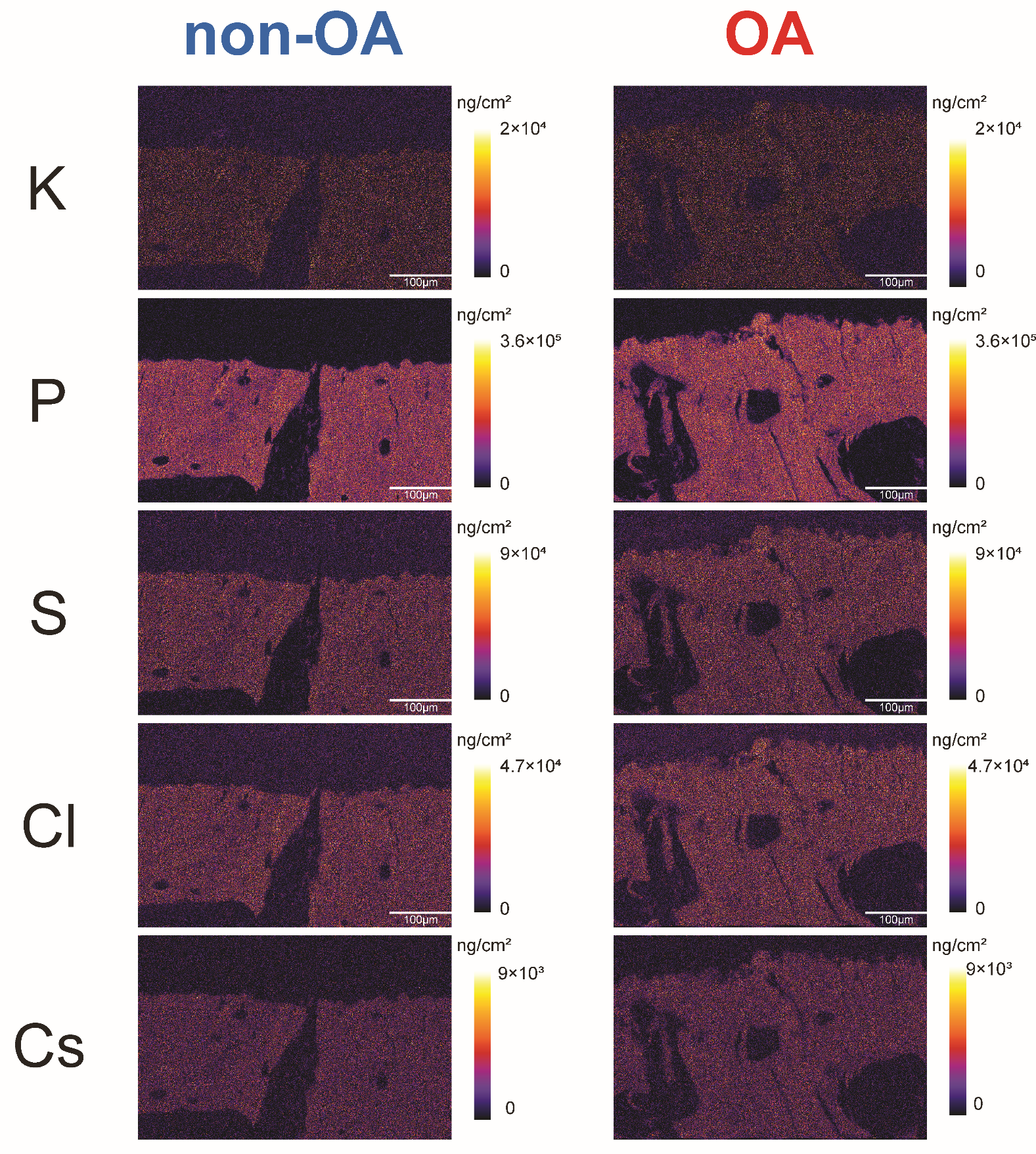
**

**Fig. S1**. **Quantitative elemental distribution of other elements in OA tissues graded according to disease severity**. Representative elemental mapping of quantitative changes between non-OA and OA osteochondral interface. Figures are representative of n=9 patient-matched samples. Scale bar: 100 μm.


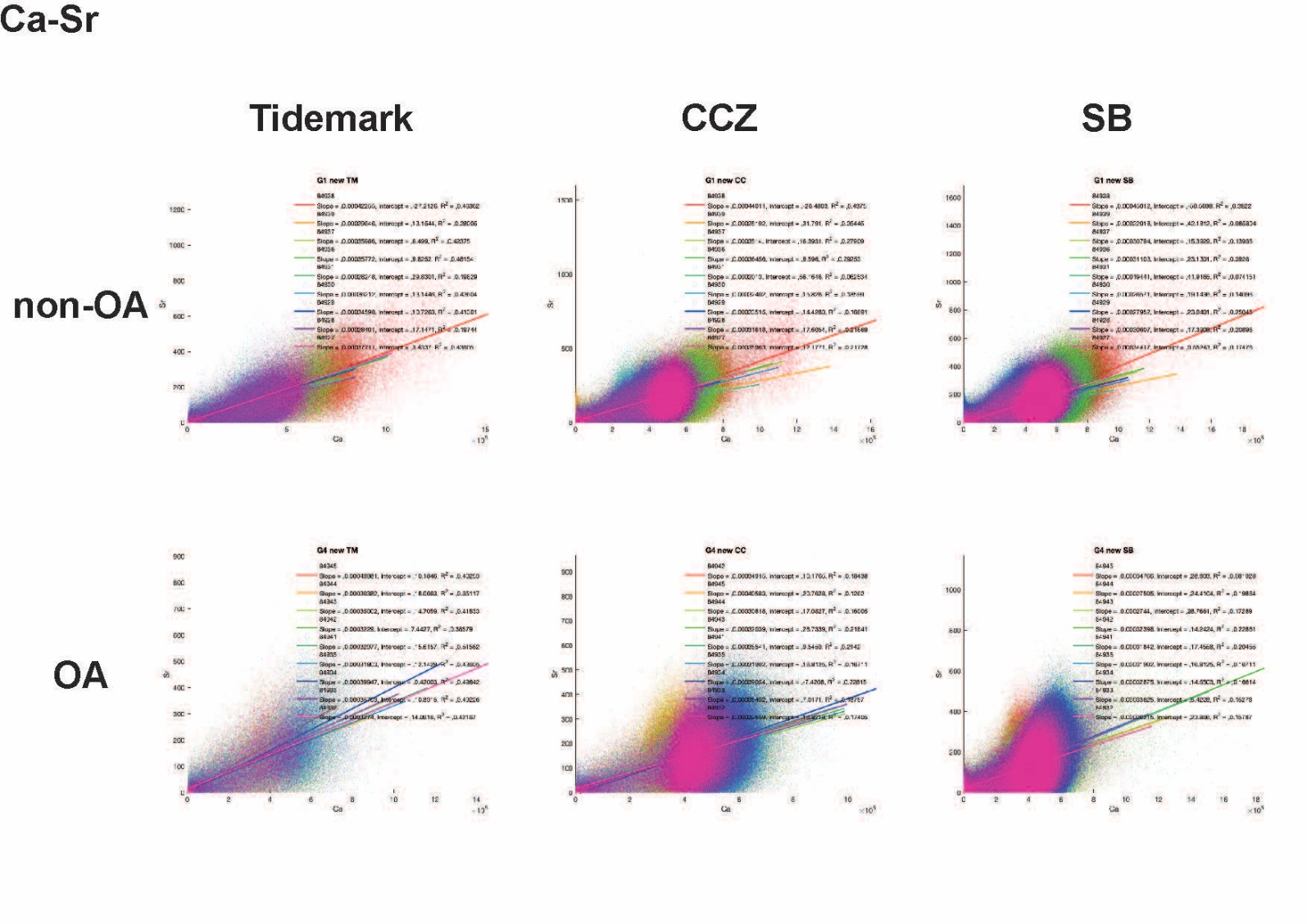
**Fig. S2**. **Quantitative Ca-Sr colocalisation analysis of osteochondral interface in OA tissues graded according to disease severity**.

**
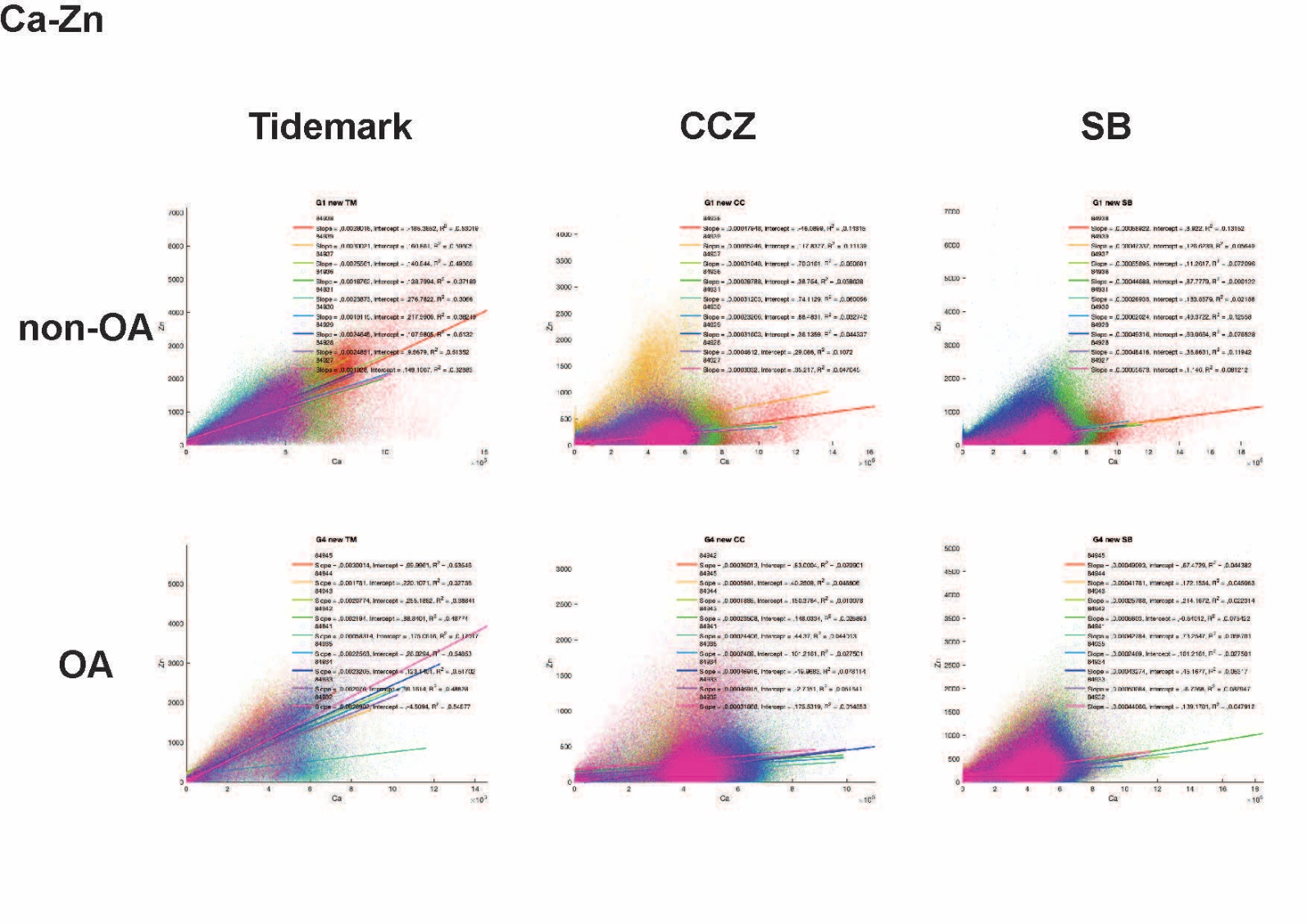
Fig. S3**. **Quantitative Ca-Zn colocalisation analysis of osteochondral interface in OA tissues graded according to disease severity**.

**
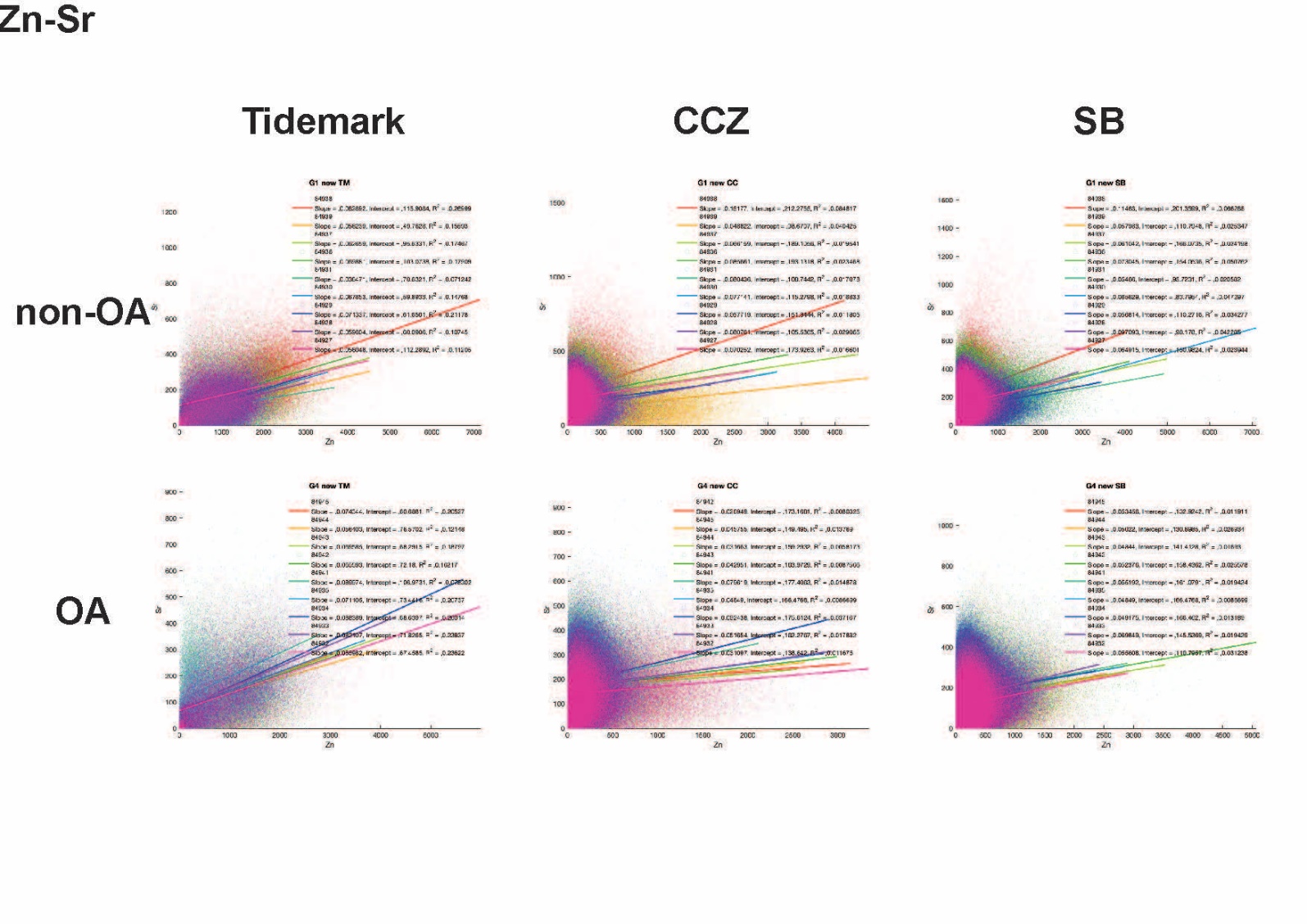
Fig. S4**. **Quantitative Zn-Sr colocalisation analysis of osteochondral interface in OA tissues graded according to disease severity**.

**
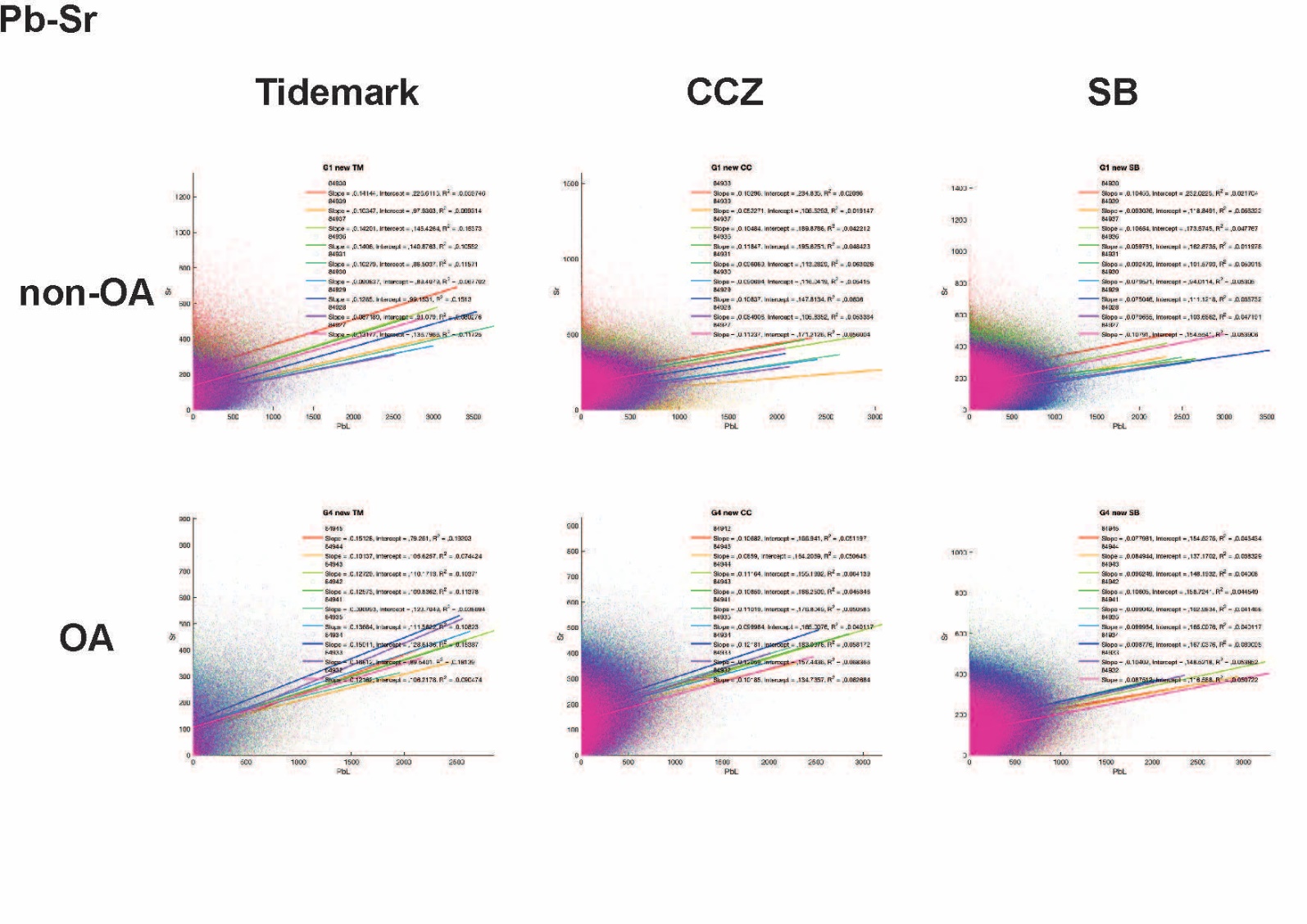
**

**Fig. S5**. **Quantitative Pb-Sr colocalisation analysis of osteochondral interface in OA tissues graded according to disease severity**.


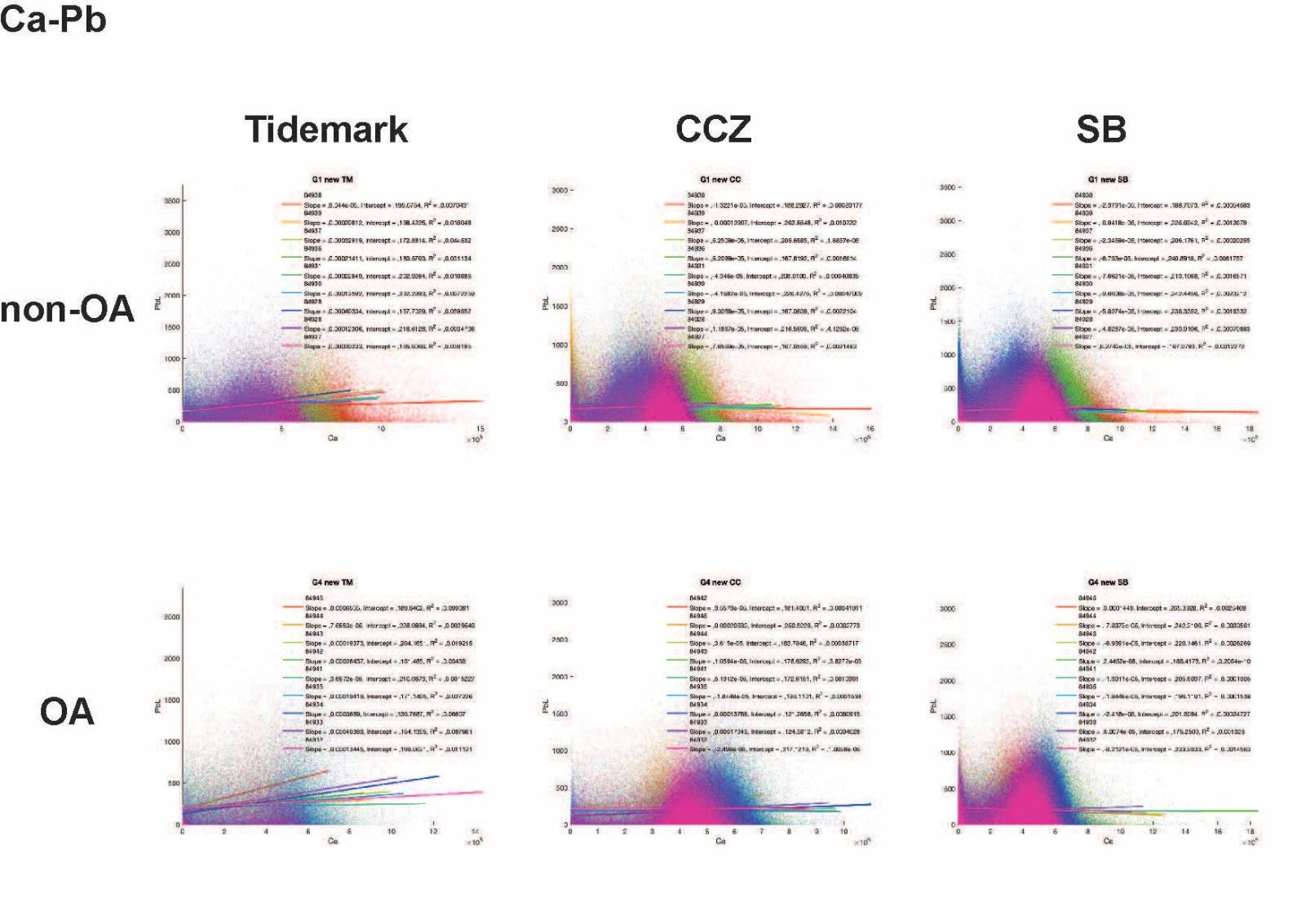


**Fig. S6**. **Quantitative Ca-Pb colocalisation analysis of osteochondral interface in OA tissues graded according to disease severity**.


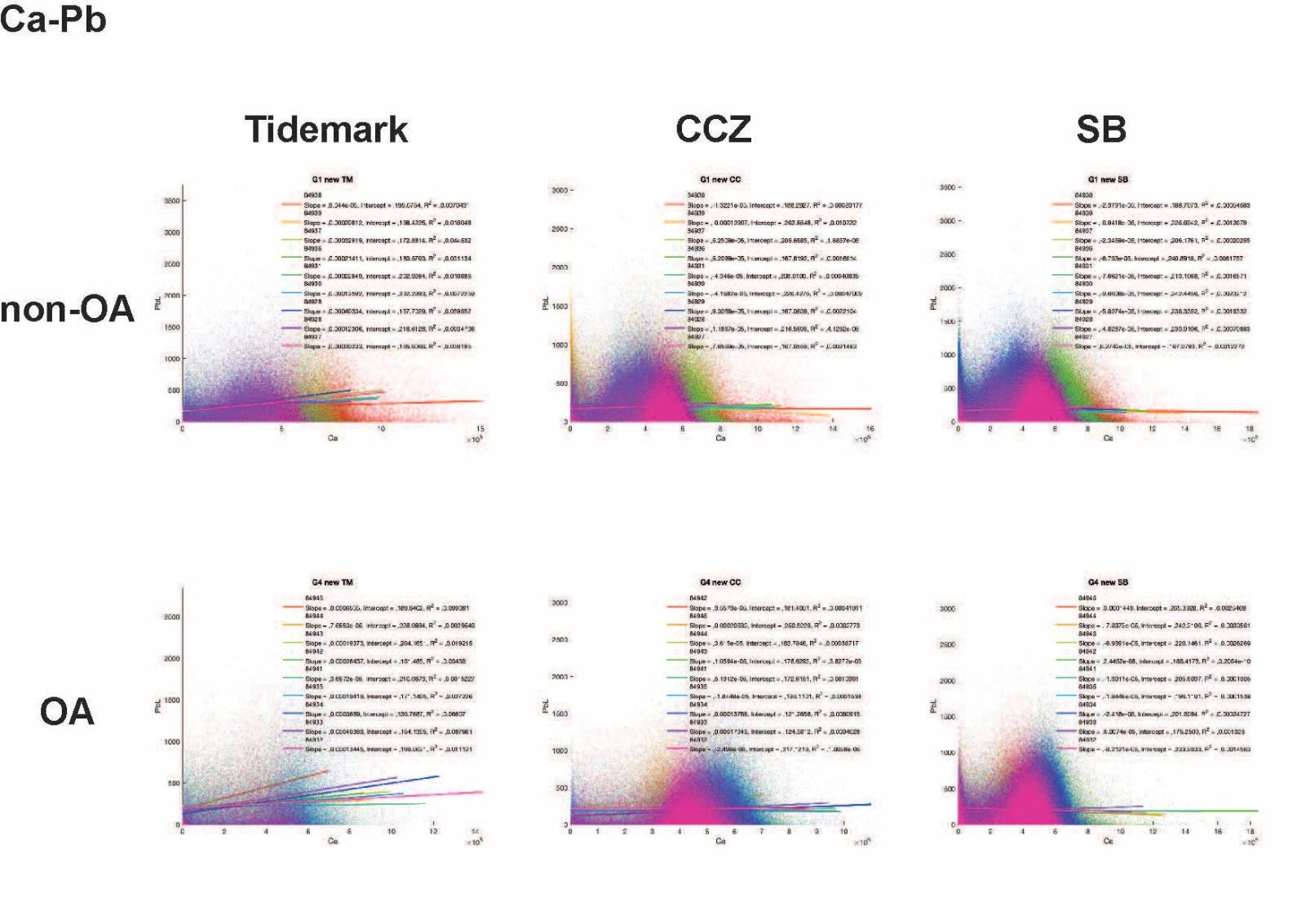


**Fig. S7**. **Quantitative Zn-Pb colocalisation analysis of osteochondral interface in OA tissues graded according to disease severity**.
