# Supplementary material for: Spatial distribution of elements during osteoarthritis disease progression using synchrotron X-ray fluorescence microscopy
Source: Sci Rep. 2023 Jun 23;13:10200. doi: 10.1038/s41598-023-36911-w (PMC10290122; doi:10.1038/s41598-023-36911-w)
Supplement: Supplementary file 2 — Supplementary Table S1. [file 41598_2023_36911_MOESM2_ESM.docx]

**SUPPLEMENTARY TABLE**

**Table S1. Demographic information of patients enrolled in the study**

| **No** | **Age** | **Gender** | **Weight (kg)** | **Height (cm)** | **BMI (kg m^-2^)** |
| --- | --- | --- | --- | --- | --- |
| 1 | 70 | Female | 73 | 158 | 29.2 |
| 2 | 73 | Female | 74 | 164 | 27.5 |
| 3 | 60 | Female | 80 | 169 | 28.0 |
| 4 | 64 | Male | 113 | 178 | 35.6 |
| 5 | 50 | Male | 88 | 163 | 33.1 |
| 6 | 71 | Female | 85 | 169 | 29.7 |
| 7 | 66 | Male | 75 | 164 | 27.9 |
| 8 | 77 | Female | 71 | 153 | 30.33 |
| 9 | 79 | Male | 85 | 169 | 29.8 |
